# Magnitude of impact and healthcare use for musculoskeletal disorders in the paediaric: a population-based study

**DOI:** 10.1186/1471-2474-13-98

**Published:** 2012-06-12

**Authors:** Anna C Gunz, Mayilee Canizares, Crystal MacKay, Elizabeth M Badley

**Affiliations:** 1 Division of Pediatric Critical Care, Children’s Hospital of Eastern Ontario, Ottawa, ON, Canada; 2 Arthritis Community Research and Evaluation Unit, Division of Health Care and Outcomes Research, Toronto Western Research Institute University Health Network, Toronto, ON, Canada; 3 Department of Physical Therapy, University of Toronto, Toronto, ON, Canada; 4 Department of Rehabilitation Science, University of Toronto, Toronto, ON, Canada; 5 Dalla Lana School of Public Health, University of Toronto, Toronto, ON, Canada

## Abstract

**Background:**

Although musculoskeletal disorders (MSD) are among the most prevalent chronic conditions, minimal attention has been paid to the paediatric population. The aim of this study is to describe the annual prevalence of healthcare contacts for MSD by children and youth age 0-19 years, including type of MSD, care delivery setting and the specialty of the physician consulted.

**Methods:**

Analysis of data on all children with healthcare contacts for MSD in Ontario, Canada using data from universal health insurance databases on ambulatory physician and emergency department (ED) visits, same-day outpatient surgery, and in-patient admissions for the fiscal year 2006/07. The proportion of children and youth seeing different physician specialties was calculated for each physician and condition grouping. Census data for the 2006 Ontario population was used to calculate person visit rates.

**Results:**

122.1 per 1,000 children and youth made visits for MSD. The majority visited for injury and related conditions (63.2 per 1,000), followed by unspecified MSD complaints (33.0 per 1,000), arthritis and related conditions (27.7 per 1,000), bone and spinal conditions (14.2 per 1,000), and congenital anomalies (3 per 1,000). Injury was the most common reason for ED visits and in-patient admissions, and arthritis and related conditions for day-surgery. The majority of children presented to primary care physicians (74.4%), surgeons (22.3%), and paediatricians (10.1%). Paediatricians were more likely to see younger children and those with congenital anomalies or arthritis and related conditions.

**Conclusion:**

One in eight children and youth make physician visits for MSD in a year, suggesting that the prevalence of MSD in children may have been previously underestimated. Although most children may have self-limiting conditions, it is unknown to what extent these may deter involvement in physical activity, or be indicators of serious and potentially life-threatening conditions. Given deficiencies in medical education, particularly of primary care physicians and paediatricians, it is important that training programs devote an appropriate amount of time to paediatric MSD.

## Background

Population data demonstrate that musculoskeletal disorders (MSD) are among the most prevalent types of chronic conditions worldwide, and are a significant cause of pain and disability [1-4]. MSD comprise a wide spectrum of conditions, including arthritis and other rheumatic disorders, as well as injury, congenital and acquired injury to the bones, soft-tissue and joints. MSD are also a major reason for physician visits [5]. However, most of the literature concerns the burden of illness in adult MSD, with relatively little attention dedicated to MSD in children.

In our previous work, we observed that almost 10% of children in the 0-15 years age group had made at least one visit to a doctor for MSD in Ontario, Canada [6]. This prompted us to look in more detail at the use of healthcare for MSD in the paediatric population, extending the analysis to include visits to paediatricians and the full range of MSD including congenital malformations. The primary aim of this study is to determine the annual prevalence of healthcare contacts for MSD in children by age group, type of MSD, care delivery setting (ambulatory care. emergency department, and hospital), as well as the specialty of the physician consulted in ambulatory care. A secondary aim is to provide information to inform priorities for medical training, future care provision and health promotion.

## Methods

The setting for this cross-sectional population-based descriptive study is Ontario, Canada which has a publicly funded healthcare system that covers all medically necessary physician visits, emergency room services, and procedures including hospital in-patient and outpatient surgery services. Access to specialists is by physician referral, with the first point of access to the medical system being the primary care or emergency room physician. Most physicians work on a fee-for-service basis: claims are submitted to provincial insurance plans for each encounter by a billing code. Some centers have salaried physicians. In this case, physicians are required to submit shadow-billing codes for all encounters to justify their salary and to ensure the employing institution receives funding from the provincial healthcare insurance plan. Therefore it is reasonable to assume that data in the physician billing system is a full record of visits to doctors by Ontario children [7,8].

We used administrative data to identify children and youth (0–19 years old) who visited physicians for MSD in Ontario in ambulatory (physician’s office) and hospital settings. The Ontario Health Insurance Plan (OHIP) database was used to capture physician office visits. Each visit was defined as one claim per diagnosis per day. The age and sex of each patient was derived by linking the data to the Ontario Registered Persons Database. The physician specialty for each visit was obtained by linking the physician billing database to the Institute of Clinical Evaluative Sciences (ICES) Physician Database (IPDB).

Children and youth who accessed hospital services were identified through the Canadian Institute of Health Information (CIHI) databases for the 2006 fiscal year (April 2006 to March 2007). These databases contain information on age, sex, and diagnostic codes. The Discharge Abstract Database (DAD) was used for hospital in-patients and the National Ambulatory Care Reporting System (NACRS) database was used for emergency visits and outpatient surgeries.

The OHIP database uses 3-digit truncated diagnostic codes, with a classification scheme adapted from the International Classification of Diseases – 9th Edition, with one code recorded for each visit. Diagnostic codes for the DAD and NACRS databases are based on diagnostic codes from the International Classification of Diseases – 10th Edition. As the DAD and NACRS databases can include multiple diagnostic codes, the diagnosis that was indicated as being the most responsible for the patient’s visit or stay in hospital was extracted.

We used the following major condition groupings: injury and related conditions (fractures/dislocations, strains/sprains); arthritis and related conditions (inflammatory arthritis, other arthritis, including osteoarthritis, soft-tissue disorders, joint derangement and unspecified arthritis); bone and spinal conditions (e.g. osteomyelitis, Legg-Calvé-Perthes disease, scoliosis); congenital anomalies (e.g. club foot, musculoskeletal anomalies) and unspecified MSD, which include ill-defined symptoms such as leg or joint pain. Diagnostic codes for each condition grouping are available in Additional file 1.

Physicians were classified as primary care physicians, paediatricians or other specialists. In Canada, paediatricians generally have referral and hospital-based practices; some provide only consultative care, and others have a mixed consultative and primary care practice [7]. Other specialists were further classified as medical or surgical. Medical specialists include rheumatologists; surgical specialists include orthopaedic surgeons. In Canada primary care for children is delivered by both primary care physicians (family physicians or general practitioners) or paediatricians [8].

### Analysis

Person visit rates to physicians in the ambulatory healthcare setting were calculated as the number of children with at least one ambulatory visit per 1,000 population. Person visit rates to hospital-based medical services were defined as the number of children with at least one hospital encounter (i.e. in-patient hospitalization, emergency department visit or outpatient surgery) per 100,000 population. In addition to this, person visit rates were calculated for each condition (e.g. inflammatory arthritis) and condition grouping (e.g. arthritis) by age and sex. All rates were calculated using census data for the 2006 Ontario population. To examine the volume of ambulatory care provided by physician specialty, the proportion of children who presented to different physician specialties was calculated by physician group and condition grouping. Children who consulted for more than one condition or who saw multiple types of physicians were counted for each condition and physician-type visited.

## Results

Overall, approximately 380,000 children and youth visited physicians in ambulatory care settings for MSD in Ontario, Canada in the fiscal year 2006/07, 122 per 1,000 children and youth aged 0-19 years (Table 1). This represents approximately 638,000 visits (1.7 visits per person per year). The musculoskeletal condition associated with each physician visit is shown in Table 1. The majority of children made visits for injury and related conditions (63.2 per 1,000), followed by unspecified MSD complaints, such as limb pain or undiagnosed conditions (33.0 per 1,000), and arthritis (27.7 per 1,000). Most of the visits for arthritis and related conditions were for soft tissue disorders and unspecified arthritis, but 1.4 per 1,000 children were coded as visiting for inflammatory arthritis. Congenital anomalies accounted for 3 per 1,000 children.

**Table 1:** Person visit rates to all physicians in ambulatory care by age and sex, Ontario, 2006/07

	**Person visit rates per 1,000 population**							**Ratio: Girls/Boys**	**Average number of visits**
	**All ages**	**Age groups**				**Sex**			
		**0-4**	**5-9**	**10-14**	**15-19**	**Girls**	**Boys**		
**All MSD**	**122.1**	**66.1**	**76.4**	**156.5**	**174.5**	**115.2**	**128.7**	**0.9**	**1.68**
**Injury and related conditions**	**63.2**	**25.2**	**39.1**	**87.8**	**91.0**	**56.0**	**70.0**	**0.8**	**1.65**
Fractures & dislocations	21.4	11.5	16.5	31.6	23.6	15.9	26.7	0.6	2.03
Strains & sprains	45.7	14.8	24.9	62.5	72.6	43.0	48.2	0.9	1.33
**Arthritis and related conditions**	**27.7**	**16.7**	**15.6**	**32.7**	**42.5**	**27.3**	**28.1**	**0.9**	**1.34**
Inflammatory	1.4	0.8	1.0	1.5	2.1	1.7	1.0	1.7	2.39
Other arthritis	2.1	1.0	0.9	2.3	4.0	2.1	2.2	0.9	1.18
Soft Tissue	11.9	3.9	5.8	15.1	20.8	11.8	12.0	0.9	1.16
Joint derangement	2.6	0.5	0.5	2.6	6.3	2.4	2.8	0.8	1.65
Unspecified arthritis	10.9	11.0	7.8	12.6	11.9	10.4	11.3	0.9	1.22
**Bone and Spinal conditions**	**14.2**	**4.8**	**6.1**	**17.6**	**25.7**	**15.1**	**13.2**	**1.1**	**1.30**
Spine	10.4	1.6	3.1	11.9	22.5	11.8	9.0	1.3	1.31
Bone	3.9	3.2	3.1	5.9	3.3	3.4	4.4	0.8	1.24
**Congenital anomalies**	**3.0**	**7.7**	**2.0**	**1.9**	**1.2**	**2.9**	**3.1**	**0.9**	**1.59**
**Unspecified MSD**	**33.0**	**18.6**	**21.6**	**41.6**	**46.4**	**31.8**	**34.2**	**0.9**	**1.22**

People with visits for more than one condition were included in all relevant groups.

For the majority of conditions, the proportion of children and youth who made ambulatory visits increased with age, with the exception of congenital anomalies that demonstrated an inverse trend. Overall more boys than girls presented to physicians with MSD complaints (girl/boy ratio 0.9), particularly for fractures and dislocations (girl/boy 0.6). A higher proportion of girls presented with inflammatory arthritis (girl/boy ratio 1.7) and spinal conditions (girl/boy ratio 1.3, respectively).

The majority of physician visits were to primary care physicians (74.4%) for all types of MSD, except congenital anomalies, where the majority of cases presented to surgical specialists (Table 2). Overall, 10.1% of children and youth saw paediatricians, 6.2% saw medical specialists and 22.3% saw surgical specialists, mainly orthopaedic surgeons. Almost half of all children with inflammatory arthritis were seen by paediatricians as well as a quarter of those with unspecified arthritis. Paediatricians only saw a minority of children with injury. Only a minority of children saw rheumatologists, mostly for inflammatory arthritis.

**Table 2:** Type of physician consulted by children with visits for MSD, Ontario, 2006/07

	**Number of children making visits to all physicians**	Percent distribution by type of physician*						
		**Primary Care**	**Paediatrics**	**All specialists**	**Medical Specialists**		**Surgical Specialists**	
					**All**	**Rheumatology**	**All**	**Orthopaedic surgery**
**All MSD**	**380,300**	**74.4**	**10.1**	**27.3**	**6.2**	**0.6**	**22.3**	**19.7**
**Injury and related conditions**	**196,750**	**69.7**	**5.0**	**33.8**	**6.0**	**0.0**	**28.3**	**26.6**
Fractures & dislocations	66,650	32.3	3.9	72.8	11.4	0.0	62.1	59.4
Strains & sprains	142,230	84.1	5.1	14.1	3.1	0.0	11.2	10.0
**Arthritis and related conditions**	**86,210**	**68.5**	**14.5**	**21.3**	**7.2**	**1.8**	**16.1**	**14.6**
Inflammatory	4,240	36.7	47.5	30.0	38.9	17.6	9.5	4.5
Other arthritis	6,680	81.5	5.7	13.5	5.2	1.5	9.8	9.0
Soft Tissue	37,100	87.0	3.4	11.3	5.4	0.5	6.5	4.7
Joint derangement	8,160	32.9	1.1	68.8	1.2	0.3	68.0	67.9
Unspecified arthritis	33,860	54.3	26.5	20.6	6.8	1.6	15.5	14.2
**Bone and Spinal conditions**	**44,140**	**69.9**	**10.9**	**23.5**	**4.0**	**0.9**	**20.8**	**19.8**
Spine	32,270	77.8	6.3	20.4	4.2	1.0	17.7	16.7
Bone	12,220	48.3	22.9	31.5	3.5	0.6	28.8	28.0
**Congenital anomalies**	**9,310**	**7.8**	**17.2**	**77.6**	**14.4**	**0.0**	**64.0**	**61.8**
**Unspecified MSD**	**102,840**	**83.1**	**11.3**	**7.5**	**3.1**	**0.4**	**4.9**	**4.2**

People with visits for more than one condition were included in all relevant groups. *Percents can add up to more than 100% as more than one type of physician could be seen.

**Figure 1:**
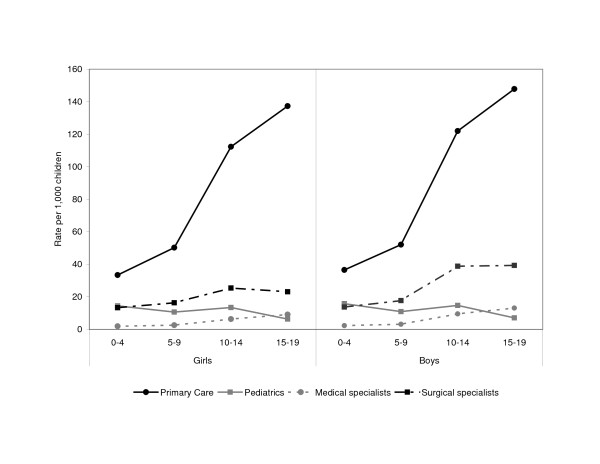
Visit rates by children with MSD in ambulatory settings by physician consulted, Ontario, 2006/07.

Figure 1 shows the age specific person visit rates by the type of physician consulted. In general, the rate of visits to medical and surgical specialists tended to increase with age. In contrast, the visit rates to paediatricians varied with age, being highest for the 0-4 years and 10-14 years age groups.

Emergency department (ED) visits for MSD conditions were made by almost 3% of children and youth in Ontario (Table 3). The most common reason for presentation was injury and related conditions, split almost equally between fractures and dislocations and strains and sprains (Table 3). Almost 20% of ED visits were for arthritis, mainly soft-tissue complaints. A minority of children and youth with MSD had admissions for outpatient surgery (137.5 per 100,000) and in-patient admissions (132.7 per 100,000). The majority of in-patient admissions were for injury (72.5 per 100,000), predominantly fractures and dislocations. Arthritis, mainly joint derangement and soft-tissue conditions, were the most common reasons for outpatient surgery.

**Table 3:** Person visit rates for MSD by hospital setting, Ontario, 2006/07

	**Person visit rate per 100,000 population**		
	**Emergency Department**	**Same Outpatient surgery**	**Inpatient**
**All MSD**	**2,627.8**	**137.5**	**132.7**
**Injury and related conditions**	**1,852.0**	**34.8**	**72.5**
Fractures & dislocations	984.6	38.4	69.1
Strains & sprains	886.7	6.1	3.4
**Arthritis and related conditions**	**450.8**	**70.9**	**32.1**
Inflammatory	7.7	1.1	10.1
Other arthritis	3.9	1.4	2.9
Soft Tissue	407.8	22.1	7.5
Joint derangement	27.9	46.1	10.7
Unspecified arthritis	6.7	0.3	1.2
**Bone and Spinal conditions**	**266.9**	**17.9**	**21.6**
Spine	220.8	0.6	3.8
Bone	47.0	17.3	17.8
**Congenital anomalies**	**0.6**	**2.8**	**1.4**
**Unspecified MSD**	**141.7**	**8.6**	**6.6**

People with visits for more than one condition were included in all relevant groups.

## Discussion

This study outlines the extent of healthcare utilization across different healthcare settings by children and youth with MSD in the population of Ontario, Canada. The most notable findings are the high proportion of children and youth who saw physicians with MSD in ambulatory care settings, and the importance of primary care physicians in assessing and triaging these patients. Our overall estimate that 1 in 8 children made physician visits each year for MSD is considerably higher than previous estimates. However, previous studies tend to base their estimates on a limited age range of patients with MSD, mainly adolescents [9,10], data from only one sector of the healthcare system, such as primary care [11], or a restricted range of diagnostic terms [11,12]. To our knowledge, this is the first study to describe the prevalence of healthcare utilization over the full age range of the paediatric population using an inclusive definition of musculoskeletal disorders and all types of physicians for a large and representative population. Our findings suggest that the prevalence of MSD in children may have been previously underestimated.

Integrating the findings from this study with an estimate for the same year that a total of 2.8 million people of all ages made physician visits for MSD [6] suggests that 13.5% of all individuals with visits to physicians for MSD were children and youth. This is a substantial proportion if one considers that generally MSD increases with age. Furthermore, if we juxtapose our findings with an estimate that 86% of children make at least one doctor visit for any condition per year in Ontario [8], it is possible of children visiting doctors for any condition that as many as 1 in 10 do so for MSD. Given this relatively high proportion it is perhaps surprising that these conditions have not received much attention in the paediatric or other medical literature.

Injury and related conditions were the most frequent reasons for contact with the healthcare system in this study. De Inocencio et. al. [13] similarly reported that injury was the most common cause of musculoskeletal pain causing children between 3 and 15 years old to present to a primary care paediatrician in an ambulatory setting in Spain. Our study found injury increases in frequency with age and was more prevalent in boys than girls, which mirrors findings from other studies [14-16]. While the underlying cause of injury is unknown, the high proportion of children with injury underscores the need to advise children and parents about safety and injury prevention. Although a minority, the number requiring surgery is of concern because this is a risk factor for longer term disability or other sequelae such as later osteoarthritis [17,18].

Overall, we found that 2.8% of children saw a physician for some kind of arthritis each year, mostly soft tissue disorders and unspecified arthritis. The nature of these conditions is unknown. The estimate of 3.5 per 1,000 children presenting with inflammatory or other arthritis is of similar order of magnitude to a US estimate [12], and our estimate for inflammatory arthritis in children of 1.4 per 1,000 is in the middle of the range of the diverse estimates in the literature [19].

While children with arthritis and related conditions were mainly seen by primary care physicians, most of children with inflammatory arthritis were seen by paediatricians. Paediatricians also saw about a quarter of children with unspecified arthritis, pointing perhaps to their more general consultative role. The relatively small proportion of children and youth with visits to rheumatologists may be an artefact of coding whereby some paediatric rheumatologists might have been coded as paediatricians in the billing database, or this could reflect existing barriers to access to paediatric rheumatologists [20]. The adult literature concerning inflammatory arthritis shows that patients who see specialists, as opposed to those who see primary care physicians, are more likely to be prescribed appropriate disease modifying anti-rheumatic drugs [21,22]. It would be interesting to know if there were treatment differences for children and youth who see different types of physicians. Visits to orthopaedic surgeons, representing 24 per 1,000 children each year, were, as expected, most frequently for injury, particularly fractures and dislocations and joint derangement.

While the majority of contacts with the healthcare system were in ambulatory settings a significant minority of children, 3%, were seen in the emergency department. Data on geographic variations in the availability of physician services in Ontario suggest that there are more emergency room visits in areas of lower physician availability, which tend to be rural and remote communities [7]. It has also been shown that children living in remote areas are likely to have longer referral times to paediatric rheumatology services [23], suggesting potential deficiencies in care for children with MSD who live in these areas.

Our study highlights the large volume of physician visits that children and youth make to primary care physicians, paediatric and other consultant specialists for MSD. There has been concern expressed about the inadequacy of training and confidence of physicians and medical students in diagnosing and managing MSD [24-26], which is not confined to adult medicine [27]. Studies in the northern regions of the UK and California in the US showed no or minimal exposure to paediatric MSD during training [27,28], and that most trainees, practicing primary care physicians and paediatricians had no or little confidence with the paediatric MSD assessment, especially in comparison with other organ systems [28]. In view of the relatively large proportion of children with MSD complaints who present to physicians, particularly primary care physicians and paediatricians, this study reinforces the need for training programs to devote an appropriate amount of time teaching residents and students about paediatric MSD conditions including how to carry out an age appropriate joint examination and to make timely referrals if warranted to a relevant specialist [23,29].

A strength of this study is that it captures all visits to physicians by children in the most populated province in Canada, as well as all emergency department visits, outpatient surgeries and inpatient hospital admissions. It shares the limitations of other studies based on administrative databases. The ambulatory care billing database uses a limited range of diagnostic codes, and the accuracy of our findings relies on the accuracy of the coding by the billing-physician. Also, as only one diagnosis can be billed for each consult, visits for MSD may be underestimated if children present with multiple conditions where MSD is not coded. Similarly, inpatient admissions may be underestimated if comorbid conditions were coded as the reason for admission. The billing data may also miss salaried physicians, although most are required to shadow-bill to OHIP, which means they would be counted in our study. There may also be inaccuracies in the recorded physician specialty, particularly between paediatricians and paediatric rheumatologists. Data on patient characteristics are also limited to age and gender with no information on other relevant aspects such as socioeconomic status or body mass index.

## Conclusion

In summary, this study provides a comprehensive overview of the extent of healthcare utilization by children with musculoskeletal disorders across different healthcare settings. To our knowledge, it is the first study of its kind to highlight the large number of children and youth, 1 in 8, who make physician visits for MSD in a year. As well as providing an overall estimate of burden of care due to MSD in children, this study may also provide prevalence estimates for specific conditions particularly those likely to be associated with healthcare intervention such as inflammatory arthritis [30]*.* While the majority of these children are likely to have self-limiting conditions, it is unknown to what extent these may deter ongoing involvement in physical activity, which is one of the cornerstones of the campaign to prevent obesity in children. This needs further research, as does the relationship between physical activity and musculoskeletal injury and potential later long-term sequelae. A further concern is that serious and potentially life-threatening conditions, including cancer as well as juvenile arthritis, may present with musculoskeletal symptoms. Deficiencies in medical education particularly of primary care physicians and paediatricians, may lead to less than optimum care for these conditions including delays in diagnosis and treatment [23,28,31]. It is therefore important that training programs devote an appropriate amount of time teaching physicians about paediatric MSD.

## Competing interests

The authors declare that they have no competing interests.

## Author’s contributions

AG collaborated in conceptualizing the project and took responsibility for drafting the manuscript. MC carried out the data extraction and analysis. CM contributed to the analysis and interpretation of the findings. EB conceived of the study and assisted in drafting the manuscript and interpretation of the findings. All authors read and approved the final manuscript.

## Pre-publication history

The pre-publication history for this paper can be accessed here:

http://www.biomedcentral.com/1471-2474/13/98/prepub

## Supplementary Material

Additional file 1 OHIP (ICD-9 based) Diagnostic Codes by Condition Groupings used in the Physician Billing Database. Click here for file
