# Supplementary material for: Magnitude of impact and healthcare use for musculoskeletal disorders in the paediaric: a population-based study
Source: BMC Musculoskelet Disord. 2012 Jun 12;13:98. doi: 10.1186/1471-2474-13-98 (PMC3493363; doi:10.1186/1471-2474-13-98)
Supplement: Additional file 1 — OHIP (ICD-9 based) Diagnostic Codes by Condition Groupings used in the Physician Billing Database. [file 1471-2474-13-98-S1.doc]

**APPENDIX 1**

OHIP (ICD-9 based) Diagnostic Codes by Condition Groupings used in the Physician Billing Database

|  | **Descriptions (OHIP)** | **OHIP diagnostic codes*** |
| --- | --- | --- |
| **Injury and related conditions** | |  |
| Fractures/dislocations | Fractures: facial bones, skull, vertebral column without spinal damage, vertebral column with spinal damage, ribs, pelvis, clavicle, humerus, radius/ulna, carpal bones, metacarpals, phalanges, femur, fibula/tibia, ankle, leg, other; Dislocations: shoulder, elbow, finger, other | 802, 803, 805, 806, 807, 808, 810, 812, 813, 814, 815, 816, 821, 823, 824, 827, 829. 831, 832, 834, 839 |
| Strains/sprains | shoulder/upper arm, elbow, wrist/hand and fingers, knee/leg, ankle/foot & toes, coccyx/neck, other | 840, 841, 842, 843, 844, 845, 847, 848 |
| **Arthritis and related conditions** | |  |
| Inflammatory arthritis | rheumatoid arthritis, Still's disease;  ankylosing spondylitis; disseminated lupus erythematosus, scleroderma, polyarteritis nodosa, temporal arteritis; pyogenic arthritis | 714, 720, 710, 446, 711 |
| Other arthritis | osteoarthritis; injurytic arthritis; gout | 715, 716, 274, 712 |
| Soft tissue disorders | synovitis, tenosynovitis, bursitis, bunion, ganglion; Dupuytren's contracture; fibrositis, myositis, muscular rheumatism | 727, 728, 729 |
| Joint derangement | joint derangement, recurrent dislocation, ankylosis, meniscus or cartilage tear, loose body in joint | 717, 718 |
| Unspecified arthritis |  | 739 |
| **Bone and spinal conditions** | |  |
| Spine | cervical arthritis, intervertebral disc disorders, pain neck, lumbar strain, lumbago, coccydynia, sciatica, lumbar disc disease (degenerative), scoliosis | 721, 722, 723, 724, 725, 737 |
| Bone | osteomyelitis, osteochondritis, Legg-Perthes disease, osteoporosis, spontaneous fracture, oth. dis. of bone & cart., flat foot, pes planus, hallux valgus, hallux varus, hammer toe | 730, 731, 732, 733, 734, 735 |
| **Congenital anomalies** | club foot, other congenital anomalies of limbs, other musculoskeletal anomalies | 754, 755, 756 |
| **Unspecified MSD** |  | 781 |
| **ICD-10 Diagnostic Codes by Condition Groupings used in NACRS and DAD databases** | | |
|  | **Descriptions (ICD-10)** | **ICD-10 diagnostic codes** |
| **Injury and related conditions** | |  |
| Fractures/dislocations | **Fractures**: neck, spine & pelvis, shoulder, elbow, hand & wrist, hip, knee & patella, ankle & foot. **Dislocations**: neck, spine & pelvis, shoulder, elbow, hand & wrist, hip, knee & patella, ankle & foot | T08,S12, S32, S42, S52, S62, S72, S82, S92. S13.0-S13.3, S33.0-S33.3, S43.0-S43.3, S53.0- S53.1, S63.0-S63.2,S73.0, S83.0- S83.1, S93.0-S93.3 |
| Strains/sprains | neck and lumbar spine, shoulder, elbow, hand & wrist, hip, knee & patella, ankle & foot | S13.4, S13.5, S33.5-S33.7 S43.4-S43.7, S53.2-S53.4, S63.3-S63.7, S73.1, S83.2-S83.7, S93.4-S93.6 |
| **Arthritis and related conditions** | |  |
| Inflammatory arthritis | rheumatoid arthritis, still's disease; ankylosing spondylitis; disseminated lupus erythematosus, scleroderma, polyarteritis nodosa, temporal arteritis | M05 - M09, M45, M46, M30-M36 |
| Other arthritis | osteoarthritis; injurytic arthritis, pyogenic arthritis; gout | M15-M19, M00-M03, M12, M10, M11, M14 |
| Soft tissue disorders | synovitis, tenosynovitis, bursitis, bunion, ganglion; disorders of muscle; fibroblastic disorders, soft tissue disorders in diseases classified elsewhere; shoulder lesions, enthesopathies lower limb; fibrositis, myositis, muscular rheumatism | M65-M71, M60-M63, M72-M73, M75-M77, M79 |
| Joint derangement | joint derangement, recurrent dislocation, ankylosis, meniscus or cartilage tear, loose body in joint | M22-M24 |
| Unspecified arthritis | other arthritis, other disorders of the musculoskeletal system | M13, M96, M99 |
| **Bone and spinal conditions** | |  |
| Spine | deforming dorsopathies, spondylosis, other dorsopathies  Spinal stenosis, ankylosing hyperostosis, other spondylopathies | M40-M43, M47, M49-M54  M48.0-M48.3, M48.8-M48.9 |
| Bone | osteopathies and chondropathies, injurytic spondylopathy; acquired deformities of fingers and toes, hallux valgus | M80-M90, M91-M94, M48.4, M48.5; M20, M21.4, L60.0, L60.2, L84 |
| **Congenital anomalies** | acquired deformities of musculoskeletal system and connective tissue | M95 |
| **Unspecified MSD** | other acquired deformities, other joint disorders | M21 excl. M21.4, M25 |
